# The effects of plyometric versus resistance training on running economy and 5‐km running time in middle‐aged recreational runners

**DOI:** 10.1002/ejsc.12197

**Published:** 2024-11-11

**Authors:** Yuuri Eihara, Kenji Takao, Takashi Sugiyama, Sumiaki Maeo, Hiroaki Kanehisa, Tadao Isaka

**Affiliations:** ^1^ Faculty of Sport and Health Science Ritsumeikan University Kusatsu Shiga Japan; ^2^ Institute of Advanced Research for Sport and Health Science Ritsumeikan University Kusatsu Shiga Japan; ^3^ National Institute of Fitness and Sports in Kanoya Kanoya Kagoshima Japan

**Keywords:** biomechanics, endurance performance, metabolism, plyometric training, resistance training

## Abstract

The effects of plyometric training (PT) versus resistance training (RT) on running economy and performance are unclear, especially in middle‐aged recreational runners. We examined (1) the efficacy of PT versus RT on running economy and performance in middle‐aged recreational runners and (2) the relationships between the main training outcomes. Twenty middle‐aged recreational runners were randomly allocated to a PT or RT group (*n* = 10/group). Training was conducted twice/week for 10 weeks combined with daily running. PT included the countermovement jump (CMJ), rebound jump, hurdle hop, and drop jump. RT consisted of leg press, leg curl, and calf raise with 50%–90% of one‐repetition maximum (1RM). Before and after the intervention, 1RM of the three lifting tasks, CMJ and drop jump performances, oxygen cost at 8–12 km/h, and 5 km running time were assessed. PT enhanced 1RM of leg curl only (8.5% and *p* = 0.007), whereas RT increased 1RM of the three lifting tasks (19.0%–21.1% and *p* < 0.001). Both groups improved CMJ height (6.4%–8.3% and *p* = 0.016) and drop jump performance (height: 9.7%–19.4%, *p* = 0.005, height/contact time: 11.4%–26.3% and *p* = 0.009) and oxygen cost regardless of running velocity (2.0% and *p* = 0.001) without significant group differences. However, neither group changed the 5‐km running time (*p* ≥ 0.259). A significant correlation was found between the changes in calf raise 1RM and oxygen cost (*r* = −0.477 and *p* = 0.046) but not between the other measured variables. These results suggest that for middle‐aged recreational runners, PT and RT can similarly improve running economy albeit not necessarily the 5‐km running time, and enhancing plantarflexion strength may particularly contribute to improving running economy.

## Highlights

- This study for the first time examined the comparative effects of plyometric training (PT) and resistance training (RT) on running economy and performance in middle‐aged recreational runners.
- The effects of PT and RT were comparable for improving running economy, but neither of them improved the 5‐km running time in middle‐aged recreational runners.
- The enhancements of plantarflexion strength induced by PT and RT were associated with the changes in running economy.
- Considering that PT is relatively easily implementable and has a lower rate of perceived exertion than RT, PT will be an alternative exercise modality to RT for improving running economy in middle‐aged recreational runners.

## 1 INTRODUCTION

The global participation in running race events has been increasing from 1.8 million in 2001 to 8 million in 2018 (Anderson, 2021). In particular, middle‐aged recreational runners (30–50 years old) account for more than half of the participation in running race events (Anderson, 2021). Success in long‐distance running is attributable to the parameters of aerobic capabilities such as running economy and maximal oxygen uptake (V̇O_2max_) (McLaughlin et al., 2010). However, it may be relatively difficult to increase V̇O_2max_ in distance runners (Midgley et al., 2006) and V̇O_2max_ gradually decreases after the age of around 30 years old (Letnes et al., 2023). On the other hand, running economy, the metabolic cost to run a given distance (Shaw et al., 2014), has been shown to be similar among runners of 20–50 years old (Dos Anjos Souza et al., 2023). In addition, improvement in running economy has been observed by adding training programs aiming to improve muscular functions (e.g., plyometric [PT] and resistance training [RT]) to daily running in a wide range of age groups (Blagrove et al., 2018; Eihara et al., 2022). However, as an adjunct to daily running, there is limited information about which types of training modalities improve running economy and performance in middle‐aged recreational runners.

As an adjunct program to daily running training, an addition of RT was shown to be more effective than running training alone in improving running economy and consequently running performance despite no change in V̇O_2max_ in middle‐aged recreational runners (Festa et al., 2019; Piacentini et al., 2013). Although these studies did not report the relationships between the main training outcomes (i.e., muscle strength, running economy, and running performance) (Festa et al., 2019; Piacentini et al., 2013), a RT‐induced gain in lower limb muscle strength would theoretically result in a lower effort relative to the load during running (Fletcher & MacIntosh, 2017), thereby improving running economy and performance, even in middle‐aged recreational runners. However, in view of practical application, the execution of RT requires specialized machines and equipment to provide resistance/loading to muscles (Whitehead et al., 2018), which may limit its accessibility for some individuals.

Compared to RT, PT can be relatively easily implemented and is suggested to decrease oxygen cost during running by enhancing the ability to utilize elastic energy, consequently contributing to improvements in running performance (Barnes & Kilding, 2015; Turner et al., 2003). Indeed, a previous study recruiting mostly young recreational runners found that the relative changes in the drop jump index (jump height divided by ground contact time) induced by jump rope training were associated with changes in a 3 km running time (Garcia‐Pinillos et al., 2020). Hence, PT can be an effective training modality as an alternative approach to RT, for improving running economy and performance in endurance runners. However, their comparative effects in middle‐aged runners remain unclear, as no study has directly compared them in this age group (or of any age group). Elucidating the comparative effects of PT and RT in middle‐aged runners will provide practical and useful information to design training programs for runners of this age and facilitate a better understanding of the associations between the main training outcomes of PT and RT.

Therefore, we compared the effects of PT versus RT, as an adjunct to daily running training, on running economy and performance in middle‐aged recreational runners and also clarified whether training‐induced changes in muscle strength and jump performance are related to those in running economy and performance. We hypothesized that PT would be similarly effective to RT in improving running economy and performance in middle‐aged runners and that changes in these variables would be related to those in muscle strength and jump performance.

## 2 METHODS

### 2.1 Participants

Twenty‐one endurance runners (7 females), who had 10.1 ± 5.2 years of running training experience, participated in the present study. Participants were asked to maintain their running training volume over the intervention period and to record their runs in a training log. Prior to the start of the present study, participants were informed of the purposes, risks, and benefits of the study and signed an informed consent form. This study was approved by the Research Committee of Ritsumeikan University (BKC‐LSMH‐2021‐086). Participants were required to meet the following inclusion criteria: (1) age of 30–50 years, (2) had not experienced periodic (≥2 days per week) PT or RT within 6 months, (3) had run half or full marathon within 2 years, (4) free from injury in the month preceding the study, and (5) had a running ≥2 days per week.

Participants were randomly allocated to either a PT (*n* = 11 and 4 females) or RT (*n* = 10 and 3 females) groups, so that there were no significant differences in age, sex, running economy, V̇O_2max_, and 5 km running time at baseline by using the packages of “dplyr” and “sampling” in RStudio (version 2022.07.1 + 554, Boston MA). One runner in the PT group dropped out of the training intervention because of an injury in his daily running training. In total, 20 participants (PT: *n* = 10, age = 41.4 ± 6.3 years, RT: *n* = 10 and age = 43.8 ± 5.4 years) completed the study (Figure S1).

### 2.2 Study design

This study used a parallel, two‐group, randomized, and longitudinal experimental design to examine the hypotheses of the present study. All participants performed the assigned training on two nonconsecutive days per week for 10 weeks. The intervention period was set to 10 weeks because recent reviews recommend a training period of 10 weeks or longer to achieve a larger improvement in running economy (Denadai et al., 2017; Eihara et al., 2022).

All participants completed the following test battery in four experimental sessions: twice before (pretest) and twice after (posttest) the training intervention. The measurements included body composition, one‐repetition maximum (1RM) of three lifting tasks, jump performance, running economy, and V̇O_2max_ on the first day and a 5 km running time trial on the second day (at least 48 h after the first day). The distance of 5 km for the time trial was chosen because (1) the 5‐km running time is one of the predictors for marathon race time (Vickers & Vertosick, 2016) and (2) in the practical field, runners often refer to a tool to predict marathon time from time trial records of shorter running distances (*Marathon Handbook*, 2016). Participants were asked to refrain from caffeine for 3 h and alcohol for 24 h before the first and second testing days and to wear the same shoes during the pre and posttest. All tests were conducted at the same time of the day (±1.5 h for each participant) to avoid the influence of the circadian rhythm.

### 2.3 Measurements

#### 2.3.1 Body composition

Height and body mass were measured with a fully automatic body scale with a stadiometer (WB‐510, TANITA Corp.). Body fat percentage and muscle mass were measured with a body composition analyzer (InBody770, InBody Japan Co. Ltd.).

#### 2.3.2 1RM

1RM was measured for leg press (HS‐SLP, Hammer Strength/Life Fitness), leg curl (Toredo, Senoh), and calf raise (IMH703, Coming Health Tech). The participants commenced a warm‐up of five repetitions at 50% of the estimated 1RM. After the warm‐up, the participants performed 3–6 trials to determine their actual 1RM by gradually increasing the load, with a rest interval of 2 min between the trials (Maeo et al., 2021). The joint angles and success criteria of each 1RM test were as follows: For the leg press, initial joint angles for the knee and ankle were both at 90° and participants sat deep in a chair. The trial was considered successful when the knees were fully extended. For the leg curl (in the prone position), the knees were first fully extended and the heel collars of the shoes were placed at the center of the cushion. When the knees were fully flexed, the trial was successful. For the calf raise, the participants stood on a calf raise machine with the hip angle at 0°, knees fully extended, and ankles fully dorsiflexed. When the participants could plantarflex to the same height as that raised without a load (recorded beforehand), the trial was considered successful.

#### 2.3.3 Jump performance

After the completion of the three 1RM tests, participants performed the countermovement jump (CMJ) and 30 cm drop jump tests three times for each test. Prior to the tests, the participants performed a warm‐up involving static or dynamic stretch exercises and each of CMJ and drop jumps three times. For the CMJ test, the participants performed a rapid downward squat movement and jumped vertically with an arm swing (Toyomura et al., 2018). Jumping heights were recorded by using an optical measurement system (Opto‐jump‐next, Microgate). For the drop jump test, participants first stood on a 30 cm height box and then stepped off the box to land and rebound‐jumped with an arm swing for maximum height with minimum ground contact time. Jumping height, contact time, and drop jump index (jump height divided by contact time) were measured using the optical measurement system. The best trial for jump height in each jump was used for data analysis. If the highest jump heights in drop jump were the same for two or three trials, we adopted the trial with the shortest ground contact time.

#### 2.3.4 Running economy and V̇O_2max_

Running economy and V̇O_2max_ were measured using a treadmill (Valiant Ultra; Lode BV) and a breath‐by‐breath gas analyzer (AE‐310s, Minato Medical Science). Heart rate was also assessed using a heart rate sensor (Polar H10, Polar). Before the test session, the gas analyzer was calibrated with gases of known concentration (16% O_2_ and 5% CO_2_). The participants warmed up on the treadmill at 8 km/h for 5 min, rested for 3 min, and then ran for 3 min at 8, 10, and 12 km/h. Running economy was calculated as the average oxygen cost (mL/kg/km) during the final minute at each running speed.

Following the submaximal running test, the participants rested for 3 min and underwent an incremental test to determine V̇O_2max_. The treadmill speed was first set to 12 km/h and then increased by 1 km/h every 1 min and then by 0.6 km/h every 1 min from 14 km/h until exhaustion, which was defined as the point when the participant could no longer run at the required velocity. The following criteria were used for the attainment of V̇O_2max_: heart rate >90% of age‐predicted maximal heart rate (208 − runners' age × 0.7) and the respiratory exchange ratio ≥1.10 (Li et al., 2019). V̇O_2max_ was determined as the highest V̇O_2_ value using a 30‐s moving window. These running protocols were performed under similar environmental conditions (22–24°C) between the pre and posttest.

#### 2.3.5 5 km running time

On the second trial day, a 5‐km running time trial was performed on a 400‐m track. The time trial was conducted at 7:00–9:00 or 18:00–21:00, with the same time zone used for the pre and posttest for each participant. Following a self‐selected 30‐min warm‐up consisting of jogging and stretching, the participants performed a 5‐km running time trial. Verbal encouragement was provided throughout the test. The 5‐km running time was recorded using a system stopwatch (SVAS011, Seiko Watch). Atmospheric temperature and humidity were measured using a digital thermo‐hygrometer (WBGT‐213B, Kyoto Electronics Manufacturing). When the temperature exceeded 28°C, we asked the participants on each lap whether they could run the next lap, to reduce the risk of heat stroke (*Ministry of the Environment, Government of Ministry of the Environment Government of Japan,* 2013). Three participants (PT group = 1 and RT group = 2) did not complete the 5‐km running time trial in the posttest based on these criteria. Thus, the sample sizes of these variables were 9 and 8 for the PT and RT groups, respectively.

### 2.4 Training intervention

Both groups performed prescribed training programs (Table 1), two sessions per week for 10 weeks with at least 48 h between the sessions. The participants were instructed to maintain regular running training but refrain from running training 6 h before conducting the added PT or RT sessions to avoid the influence of fatigue and potential interference effects (Fyfe et al., 2014; Garcia‐Pallares et al., 2009). The PT and RT programs were conducted according to their group allocation. All training sessions were supervised. At the end of each training session, we assessed the rate of perceived exertion (RPE) for the legs and whole body using the Borg CR10 scale (Williams, 2017).

**TABLE 1:** Training programs in PT and RT.

	Week 1–2	Week 3–4	Week 5–6	Week 7–8	Week 9–10
PT group					
CMJ	3 sets × 8–10 reps				
3RJ	6–8 sets				
BL hurdle hop	3 sets × 8–10 reps	4 sets × 8–9 reps	4 sets × 10 reps		
20 cm DJ		3 × 8–9 reps	3 sets × 10 reps		
5RJ		4–5 sets	6 sets	6–7 sets	8 sets
SL hurdle hop				4 sets × 8–9 reps	4 sets × 10 reps
30 cm DJ				3 sets × 8–9 reps	3 sets × 10 reps
RT group					
Leg press, leg curl, and calf raise	50%–60% 1RM	75% 1RM	80% 1RM	85% 1RM	90% 1RM
	3 sets × 10 reps	3 sets × 8 reps	3 sets × 6 reps	3 sets × 5 reps	3 sets × 4 reps

Abbreviations: 1RM, one repetition maximum; BL, both legs; CMJ, counter‐movement jump; DJ, drop jump; PT, plyometric training; RJ, rebound jump (e.g., 5RJ: 5 rebound jump); RT, resistance training; SL, single leg.

For PT, the participants were instructed to jump explosively with minimum ground contact time (except for CMJ) and maximum jump height. Ground contact time was monitored using a mat switch platform system (Multi jump tester, DKH), and instant feedback was given to the participants to conduct jumps with a ground contact time of ≤300 ms. For RT, the training was performed without countermovement actions, taking 2 s for each of the concentric and eccentric phases, under the guidance of a metronome (60 bpm). Two‐minute rest intervals were taken in between sets.

### 2.5 Statistical analyses

Twenty runners (10 per group) completed more than 90% of their prescribed training sessions and were included in the analysis. Descriptive data are presented as means and standard deviations. Statistical analyses were performed using RStudio (version 2022.07.1+554). Statistical significance was set at *p* < 0.05. The normality of distribution was tested using Shapiro–Wilk's test and all variables were confirmed as normally distributed. The RPE of training was averaged across all training sessions and the group differences were tested using an independent *t*‐test. Regarding the variables measured at the pre and posttest, all except for oxygen cost of running was tested using a linear mixed model (group and time). When a significant group‐by‐time interaction was found in a linear mixed model, (1) a paired *t*‐test was performed for the pre and posttest values within each group and (2) absolute change values were calculated and compared between groups by an unpaired *t*‐test as post hoc tests. For oxygen cost of running, a repeated 3‐way ANOVA (group, time, and running velocity) was performed. Hedges' *g* values were calculated to estimate the magnitude of changes in outcomes between the pre and posttest in each group and were interpreted as trivial ≤0.20; small 0.20–0.50; moderate 0.50–1.00; and large ≥1.00 (regardless of its sign, negative or positive (Cohen, 1988)). To improve the statistical inference, the mean difference from baseline with a bootstrap 95% confidence interval (5000 samples, bias‐corrected, and accelerated) was calculated for each of the main training outcomes (1RM, jump, and aerobic parameters) by using the estimation statistics (Ho et al., 2019). In addition, within‐participant repeated measures correlations were performed using the R package “rmcorr” to assess the common intraindividual association between changes in the main training outcomes (Bakdash & Marusich, 2017).

## 3 RESULTS

There were no significant differences between the two groups (by independent *t*‐tests) in all variables at baseline. Anthropometric, physiological, and running data are shown in Table 2. The average of RPE across all sessions was significantly lower for the PT group (leg: 3.5 ± 1.4 and whole body: 3.4 ± 1.1) than the RT group (leg: 5.7 ± 1.6, whole body: 4.7 ± 1.9 and *p* < 0.001 for both). There were no significant changes in height, body mass, body fat percentage, muscle mass, and V̇O_2max_ in both groups (*p* = 0.079–0.896, Table 2). Weekly running training volume had a significant main effect of time (PT: 44.8 ± 22.8 km/week to 43.1 ± 18.6 km/week, RT: 37.5 ± 1.1 km/week to 28.5 ± 1.0 km/week, and *p* = 0.010) with no significant main effect of the group (*p* = 0.124) and interaction (*p* = 0.117), indicating that both groups reduced the running training volume during the intervention period compared to before the intervention.

**TABLE 2:** The changes of characteristics of participants from pre‐ to post‐intervention.

	PT group (*n* = 10)			RT group (*n* = 10)		
	Pre	Post	*g*	Pre	Post	*g*
Height (m)	1.68 ± 0.10	1.68 ± 0.10	0.02	1.67 ± 0.08	1.67 ± 0.08	0.01
Body mass (kg)	61.4 ± 14.2	61.6 ± 14.7	0.01	58.3 ± 9.3	58.4 ± 9.5	0.01
Body fat percentage (%)	18.2 ± 5.4	17.3 ± 6.0	−0.15	18.0 ± 6.6	17.0 ± 5.4	−0.16
Muscle mass (kg)	46.9 ± 9.5	47.6 ± 10.2	0.07	45.1 ± 8.8	45.7 ± 8.7	0.07
$\dot{V}O_{2\;\max}$ (mL/kg/min)	46.5 ± 8.2	46.0 ± 6.3	−0.08	47.3 ± 6.7	47.3 ± 5.8	−0.04

*Note*: The values were mean ± SD. Hedges' *g* was calculated from the change from pre to posttest.

Abbreviations: PT, plyometric training; RT, resistance training.

**FIGURE 1:**
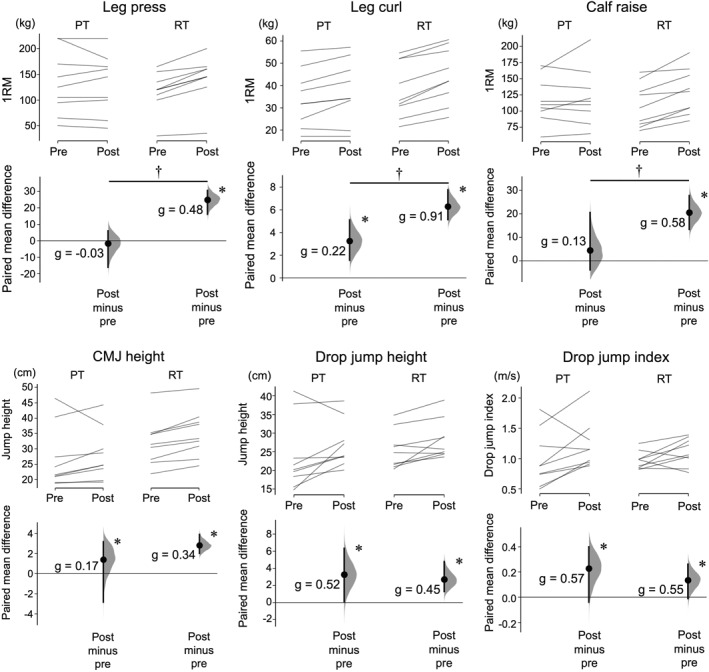
Changes in 1RM of the three lifting tasks and jump performances after the training in the PT and RT groups. In each subfigure, the raw data are plotted on the upper axes for PT (left) and RT (right); each paired set of observations at the pre and posttest is connected by a line. On the lower axes, each paired mean difference is plotted as a bootstrap sampling distribution. Mean differences are depicted as dots with horizontal dashed lines; 95% confidence intervals are indicated by the ends of the vertical error bars. Hedges' *g* represents the magnitude of the changes in the variables from pre to post. *Significant change from pre to post (*p* < 0.05). ^†^Significant difference between groups (*p* < 0.05). 1RM one repetition maximum; PT, plyometric training; RT, resistance training.

Significant group‐by‐time interactions were found in all 1RM tests (*p* = 0.001–0.017). PT significantly increased the 1RM of leg curl (*p* = 0.007) but not leg press and calf raise (*p* = 0.372–0.774), whereas RT significantly increased the 1RM values of the three lifting tasks after the intervention (*p* < 0.001, Figure 1). Significant main effects of time were observed for all jump performance variables (*p* = 0.005–0.016) without group‐by‐time interactions (*p* = 0.248–0.603, Figure 1), indicating significant increases in all jump performances for both groups, without a significant group difference. These results were the same when the 1RM values were normalized to body mass.

**FIGURE 2:**
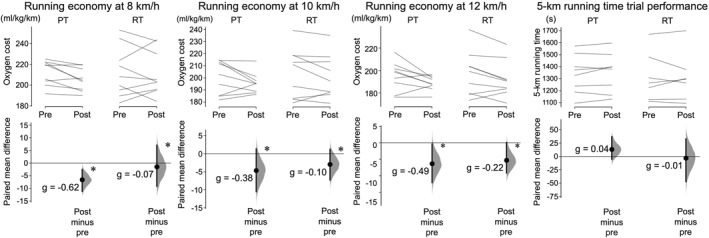
Changes in oxygen cost of running (running economy) at 8, 10, 12 km/h, and 5‐km running time after the training in the PT and RT groups. In each subfigure, the raw data are plotted on the upper axes for PT (left) and RT (right); each paired set of observations at the pre and posttest is connected by a line. On the lower axes, each paired mean difference is plotted as a bootstrap sampling distribution. Mean differences are depicted as dots with horizontal dashed lines; 95% confidence intervals are indicated by the ends of the vertical error bars. Hedges' *g* represents the magnitude of the changes in the variables from pre to post. *Significant change from pre to post (*p* < 0.05). PT, plyometric training; RT, resistance training.

A repeated 3‐way ANOVA regarding oxygen cost of running showed a significant main effect of time and running velocity (*p* = 0.001 and 0.008, respectively), without a main effect of group or any interactions (*p* = 0.234–0.881, Figure 2), indicating that both groups similarly reduced oxygen cost (improved running economy) regardless of running velocity. No significant main effects or group‐by‐time interactions were detected in the 5 km running time trial performance (*p* = 0.491–0.745, Figure 2). A significant correlation was found between training‐induced changes in calf raise 1RM normalized to body mass and oxygen cost of running (averaged across 8–12 km/h, *r* = −0.477, *p* = 0.046, and Table 3) but not between the other measured variables (*r* = 0.069 to −0.384 and *p* = 0.162–0.953).

**TABLE 3:** The correlation coefficients between the changes in aerobic working capacities (oxygen cost of running averaged across 8–12 km/h and 5‐km running time) and muscular function (1RM normalized to body mass and jump performances).

	Leg curl 1RM	Leg press 1RM	Calf raise 1RM	CMJ height	DJ height	DJI
Oxygen cost of running						
*r* (95% CIs)	−0.384 (−0.721 to 0.101)	−0.196 (−0.608 to 0.298)	−0.477 (−0.772 to −0.012)	−0.063 (−0.514 to 0.416)	0.074 (−0.407 to 0.523)	0.224 (−0.270 to 0.626)
*p* value	0.162	0.434	0.046*	0.805	0.769	0.370
5‐km running time						
*r* (95% CIs)	−0.168 (−0.589 to 0.324)	−0.323 (−0.686 to 0.170)	0.069 (−0.411 to 0.519)	0.015 (−0.455 to 0.478)	−0.041 (−0.499 to 0.434)	−0.095 (−0.538 to 0.389)
*p* value	0.505	0.192	0.784	0.953	0.870	0.708

Abbreviations: 1RM, one repetition maximum; CMJ, countermovement jump; DJ, drop jump; DJI, drop jump index.

*Significant correlation between variables (*p* < 0.05).

## 4 DISCUSSION

To the best of our knowledge, this is the first study to directly compare the effectiveness of PT versus RT, as an adjunct to daily running training, on running economy and performance in middle‐aged recreational runners. One of our main findings was that PT was similarly effective to RT in improving running economy, although none improved 5 km running time. These results partly supported our first hypothesis. In addition, training‐induced changes in calf raise 1RM were correlated with those in running economy, which also at least partly supported our second hypothesis. Collectively, these results suggest that PT and RT are similarly effective in improving running economy in middle‐aged recreational runners, with training‐induced changes in muscular function potentially translating into improvements in running economy.

In the present study, PT significantly increased CMJ height and drop jump performances and RT increased 1RM of the three lifting tasks and jump performances (Figure 2). This is reasonable based on the training specificity of both training modalities (Suchomel et al., 2018) and some transferring effects of RT (i.e., RT can improve jump performance (Damasceno et al., 2015)). Several previous studies suggest that increasing muscle strength and jump performance would improve long‐distance running performance (Barnes & Kilding, 2015; Blagrove et al., 2018; Kyröläinen et al., 2001). The present study found a significant relationship between the training‐induced changes in the calf raise 1RM and running economy. Dorn et al. (2012) found that the triceps surae has a ∼75% contribution to the vertical support impulse to accelerate the body upward during running. In addition, Willer et al. (2021) reported that the plantar flexors showed the highest activity among lower limb muscles in the propulsion phase during running and suggested that its fatigue resistance may be the key for better running performance. Furthermore, PT and RT were shown to enhance the stiffness (Spurrs et al., 2003) and musculotendinous properties (Albracht & Arampatzis, 2013) of the triceps surae and the Achilles tendon, thereby improving running performance although we did not measure these parameters. Therefore, of the lower‐limb muscles, enhancing the functions of plantar flexors may have a particularly critical role in improving running economy.

We found significant improvements in running economy for both PT and RT (Figure 2). In particular, the improvement in running economy induced by PT (2.0%–3.4% and effect size: *g* = −0.38 to −0.62 “small‐to‐moderate”) was larger than that in our recent review (<1% and *g* = −0.13 [95% confidence intervals −0.47 to 0.21]) (Eihara et al., 2022). Additionally, the improvement in running economy in the PT group may be meaningful because the margin of improvement in running economy has been reported as 2.4% (Barnes & Kilding, 2015). Two potential explanations can be made for the meaningful versus trivial effects of PT found in the current study compared to previous studies. First, recent studies have suggested that setting a longer training period for PT and RT facilitates improvements in running economy (Denadai et al., 2017; Eihara et al., 2022). Thus, we conducted PT in the current study for 10 weeks, which is longer than the training periods used in most previous studies (see the review (Eihara et al., 2022)). Second, the jump training is advised to be performed with a ground contact time comparable to that during running (i.e., 200–300 ms (Dorn et al., 2012)). Therefore, we monitored the ground contact time during jump training and the participants in the PT group of the present study could perform jumps within 300 ms of the ground contact time in 98% of the training sessions. Furthermore, the RPEs during the training for the leg and whole body were both significantly lower in the PT group than in the RT group, which is practically of great importance. Collectively, PT can be considered a relatively easily implementable yet similarly effective training modality, compared to RT, for improving running economy in middle‐aged recreational runners (Damasceno et al., 2015).

On the other hand, neither group improved 5 km running time despite the improvement in running economy. This may be partly because running performance was measured only in the 5 km time trial condition, resulting in the mismatch of the running velocity between the measurements of running economy and running performance. More specifically, the average running velocity during the 5 km running time trial in this study was 13.9 km/h, which is higher than the velocities (8–12 km/h) at which running economy was measured in this study. This difference may be a reason for the lack of significant improvement in running performance despite significant improvements in running economy. In other words, running economy might have a stronger relationship with/effect on running time trial performance over longer running distances (with lower velocities) as suggested by previous studies (Abad et al., 2016; Dellagrana et al., 2015; Fay et al., 1989; Festa et al., 2019; Nummela et al., 2006). Hence, we recommend that future studies measure running economy and performance over a wider range of distances/running velocities so that the effects of training intervention on running economy and performance, as well as the relationships between them and other training outcomes, can be examined in more detail.

It may be of practical relevance that both training groups reduced their running volume during the training intervention even though we asked the participants to maintain their daily running volume. This is likely because it was challenging for them to maintain the running volume while coming to the university to participate in the training intervention (PT: 20.2 ± 1.5 min/session and RT: 23.9 ± 1.1 min/session) in combination with their daily working schedules. However, previous studies have found that RT in parallel with running training was successful in improving running economy and 10‐km running time despite a 40%–58% reduction in the daily running volume (Skovgaard et al., 2014; Vorup et al., 2016). We also retrospectively assessed the training effects on running economy and 5 km running time using ANCOVA while accounting for running volume, but did not detect any significant effects. Thus, we consider that a reduction in running volume did not significantly influence our main findings. Nevertheless, running training programs should also be carefully monitored and adjusted in future studies to better understand their interaction with RT and/or PT and consequently enhance running performance.

## 5 CONCLUSIONS

As an adjunct exercise program to daily running training in middle‐aged recreational runners, not only RT but also PT was found to be effective in improving running economy. However, this did not lead to improving 5‐km running performance in either training group and further research focusing on longer‐distance performance is warranted. It is practically important that PT can be performed with lower perceived fatigue than RT and does not require specialized machines and equipment. In terms of feasibility in combination with daily running training, therefore, PT will be an alternative exercise modality to RT for improving running economy in middle‐aged recreational runners. Finally, a significant correlation was found between the training‐induced enhancements in calf raise 1RM and running economy but not between the other measured variables, suggesting a key role of the plantar flexors for economic running.

## CONFLICT OF INTEREST STATEMENT

The authors declare that they have no conflicts of interest. The results of this study are presented clearly, honestly, and without fabrication, falsification, or inappropriate data manipulation.

## Supporting information

Figure S1

## Data Availability

The data that support the findings of this study are available from the corresponding author upon reasonable request.
