# Supplementary figures and images for: The effects of plyometric versus resistance training on running economy and 5‐km running time in middle‐aged recreational runners
Source: Eur J Sport Sci. 2024 Nov 11;24(12):1820–9. doi: 10.1002/ejsc.12197 (PMC11621386; doi:10.1002/ejsc.12197)

**
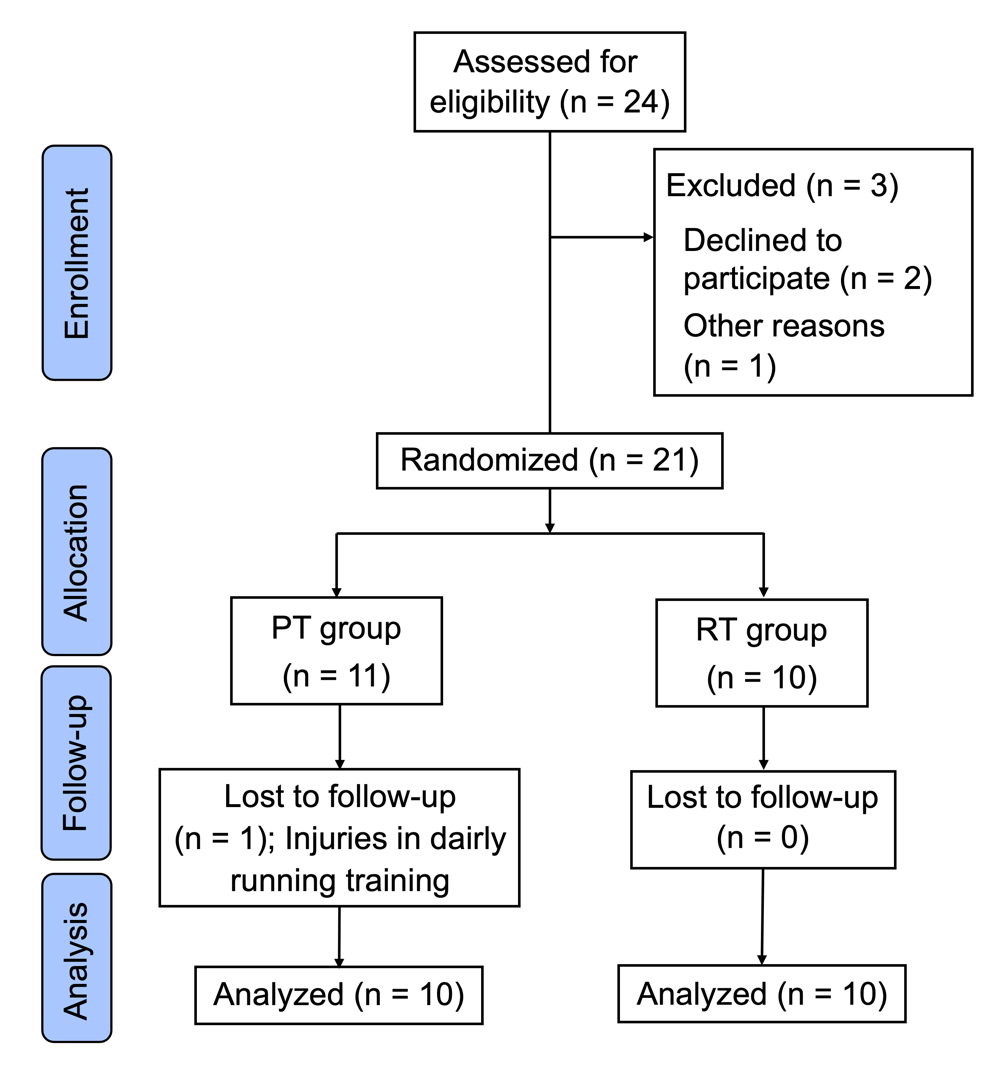
**

**Supplementary 1.** CONSORT diagram of the full recruitment and randomization process.

Supplement: Supplementary file 1 — Figure S1 [file EJSC-24-1820-s001.docx]
